# Impact of Facebook on Glucose Control in Type 1 Diabetes: A Three-Year Cohort Study

**DOI:** 10.2196/diabetes.7693

**Published:** 2017-06-07

**Authors:** Goran Petrovski, Marija Zivkovic

**Affiliations:** 1 University Clinic of Endocrinology and Diabetes Center for Insulin Pump and Sensor University Ss. Cyril and Methodius Skopje The Former Yugoslav Republic Of Macedonia

**Keywords:** social media, type 1 diabetes, insulin pump, Facebook

## Abstract

**Background:**

As the world is changing, traditional health care services should be adapted for the new era of technology and the Internet. One of the possible ways for communication between health care providers and patients is social media. There are several benefits of social media in health: increased interactions with others; more available and shared information; increased accessibility; social or emotional support.

**Objective:**

The aim of this study was to evaluate the results of Facebook and CareLink software as a possible Internet tool to improve diabetes control in type 1 diabetes patients using a sensor augmented pump.

**Methods:**

A total of 67 adolescents with type 1 diabetes and in the age range of 14-23 years were randomized in 2 groups: (1) Traditional group and (2) Internet group. In the traditional group, 34 patients were treated using standard medical protocol with regular clinic visits, where data were uploaded at the clinic and interventions (pump settings-basal bolus insulin and education) were delivered to the patient. In the Internet group, 33 patients were treated using Facebook and CareLink software (Medtronic Diabetes) on a monthly basis, where the data were uploaded by the patient at home and interventions (same as traditional group) were delivered via Facebook (written reports and chats).

Both the traditional and Internet group had regular visits every 3 months with standard medical protocol. Glycosylated hemoglobin (HbA1c) was obtained before and every 3 months during the study for a 3-year-period.

**Results:**

The improvement in glucose control was found in both groups: 7.9% (SD 1.4) [62.8 mmol/mol (SD 12.9)] to 6.9% (SD 1.2) [51.9 mmol/mol (SD 10.8)] in the traditional group, and 7.8% (SD 1.8) [61.7 mmol/mol (SD 17.2)] to 6.7% (SD 1.8) [49.7 mmol/mol (SD 17.3)] in the Internet group). Significant improvement of HbA1c (*P*<.05) was found in favor of the Internet group.

**Conclusions:**

Social media such as Facebook as a tool can assist in standard medical care to improve glucose control in a long term period in adolescents with type 1 diabetes using insulin pump therapy.

## Introduction

Diabetes is a public health problem of increasing magnitude. The prevalence of diagnosed diabetes increased by 49% from 1990 to 2002 and is expected to increase by 165% from 2000 to 2050 [1]. Health care providers are faced with an increased need of services with enormous number of patients and visits. As the world is changing, traditional health care services should adapt to the new era of technology and the Internet.

Patients use the Internet to seek, meet, and interact with a community of patients with similar problems to share clinical information and to provide and receive support [2-4]. This is a new type of dynamic online communication in contrast to earlier health-related websites. It offers patients an opportunity to benefit from a social media to learn about their illness and to gain support from others with similar experiences.

Patients are becoming increasingly active on the Web [5]. One study reports that searching for health care information was the third most common Web-based activity [6], whereas the other study reports that 72% of adult Internet users search for support and medical information on the Web [7]. Other studies demonstrate that 67% of Internet users were using social media for whichever purpose, whereas 26% were using it for health-related issues [8].

One of the possible ways to deliver Internet care is social media, which can be deﬁned as a group of Web-based applications that allow for the creation and exchange of user-generated content [9].

There are several benefits [10] of using social media in health: increased interactions with others, increased availability and sharing of information; increased accessibility; and peer, social, or emotional support. But there are also some limitations: lack of reliability, quality concerns, lack of confidentiality and privacy, risks of disclosing personal information on the Web, and harmful or incorrect advice.

Social media is sometimes viewed as manipulative and often perceived as a contradiction in terms because it is often interpreted as the business of selling goods and services. On the other hand, it can be used as a social purpose for behavior change and improved health [11].

There are different types of social media and they can overlap among the various services. With over 1.78 billion active monthly users worldwide in 2016 [12], Facebook is an important Web-based meeting place for social networking. Many specific groups for disease management have arisen on Facebook, representing important sources of information, support, and engagement for patients with chronic disease. However, relatively little research has been conducted for disease management. Facebook and Facebook groups serve as promotional spaces, support patients and their families [13], are repositories of recruitable research subjects, and serve as venues for the solicitation and provision of forms of disease management knowledge not necessarily available through more formal channels of professional consultation [14]. Recent studies evaluate the data of Facebook groups, where the mothers of children with type 1 diabetes seek and provide information to better manage the disease’s daily demands [15] with 5 dominant thematic clusters (“*food and correction,*” “*diabetes and life,*” “*hi group,*” “*bureaucracy,*” and “*needle*”). The data from discussion boards with use of computer technology can assist health care providers to address these problems and improve glucose control and quality of life [16]. Facebook group “Diabetes Macedonia” was formed by patients’ needs in 2008. Its first task was to share diabetes information among patients. It is a closed group that helps patients to communicate and share their experience with other patients. The enormous growth of new users (1840 patients, family members, etc, by September 2012) led to the creation of a structured platform by health care providers (doctors and nurses) to adjust and correct the information posted by patients, if needed.

The aim of the study was to evaluate results of Facebook and CareLink software as a possible Internet tool to improve diabetes control in adolescents with type 1 diabetes on sensor augmented pump. To our knowledge, this is the first long-term study where Facebook is used as a supplemental treatment to traditional clinic visits.

## Methods

Study participants included adolescents with type 1 diabetes, aged 14-23 years, with diabetes duration of 6.1 (2.3) years, treated with an insulin pump and sensor for at least 6 months, and had at least two outpatient visits to our center in the past year with intention to return. We did not find significant difference of glycosylated hemoglobin (HbA_1c_) level in the beginning of the study with 6 months before entering the study.

Patients were recruited at their regularly scheduled appointments, where the adolescent met with a trained research assistant who obtained written informed consent and assent, respectively. All procedures followed were in accordance with the ethical standards of the responsible committee on human experimentation (institutional and national) and with the Helsinki Declaration of 1975, as revised in 2005. Eligible patients and families were sequentially approached until 70 agreed to participate (3 patients did not finish the study).

Patient’s participation was on a voluntary basis, and the opportunity to join the Facebook group “Diabetes Macedonia” was promoted in our service with an appropriate information packet. Patients were randomized in 2 groups:

- Traditional group: A total of 34 type 1 diabetes patients were treated using standard medical protocol with regular visits at clinic, where the data were downloaded using CareLink professional software (Medtronic Diabetes) at the clinic, and intervention (education, pump settings, basal and bolus insulin) were delivered to the patient.
- Internet group: A total of 33 type 1 diabetes patients were treated using CareLink personal program (Medtronic Diabetes), where the data were uploaded by the patient at home, automatically transferred to CareLink Professional, and interventions (same as traditional group) were delivered via Facebook (written reports and chats).

Insulin pump therapy was performed using aspart, lispro, and glulisine in a Medtronic insulin pump (model 722 or Veo) with a Medtronic MiniLink sensor. En-lite sensors (Medtronic Diabetes) were used on a continuous basis during the study and patients were encouraged to use the sensor at least 80% of the time. A CareLink personal account was prepared for all the patients and linked to the CareLink Professional software, which was used to analyze diabetes control.

During the entire study, all the patients received a standardized protocol of education about correct diabetes control provided by diabetologist and diabetes nurse, including carbohydrate counting, balanced nutritional program, and regular physical activity (3 h a week).

Patients from both groups were members of the Facebook group, and all of them got an opportunity to share their experience. Health care providers followed the posts on Facebook and intervened if anyone needed further information and advice. This information was available for patients from both groups. The traditional and Internet group had the same intervention delivered by different methods retrospectively (in clinic visit vs Facebook visit).

The duration of visit was 22 min (SD 4.5) in the traditional group and 21 min (SD 3.2) in the Internet group. The communication in the Internet group was performed using Facebook messages and chats.

Internet visits were mostly performed by physicians, and changes in the pump (ICHR, ISF, basal rates, and bolus wizard) was done by patients, if needed. Advice from nurses was mainly on support and technical aspects (low and high blood sugar, regular SMBG, infusion rotation, tubing, etc).

The following characteristics were evaluated (self-reports and previous medical history): age, duration of diabetes, and body mass index. Severe hypoglycemic events (defined as glucose level <2.8 mmol/l and inability to self-treat, requiring treatment by another person, where glucagon or intravenous glucose was required to solve the situation), and diabetic ketoacidosis (DKA) episodes (defined as hospital admission due to ketoacidosis with positive ketonemia or ketonuria, hyperglycemia >11 mmol/L and pH <7.3, and clinical signs with episodes of hyperglycemia) were also evaluated during the study.

Hb_A1c_ (by high-performance liquid chromatography, reference value 4.6-5.8% [26.8 mmol/mol (SD 39.9)]) was measured every 3 months during the study in the 3-year-period. Mean blood glucose and insulin requirements were obtained from CareLink software every 3 months. Weight was measured at the beginning and at the end of the study.

Statistical analysis was performed with SAS version 8 for Windows (SAS Institute). Frequency distributions and appropriate summary statistics for central tendency and variability were used to describe possible differences between the two groups. The analyses included paired *t* tests to compare potential differences in Hb_A1c_ between two groups from baseline.

## Results

Clinical characteristics are shown in Table 1. There was no significant difference of patient’s characteristics.

All patients had Hb_A1c_ above 7.5% (58.5 mmol/mol) before enrolling in the study. Hb_A1c_ decreased in both groups: 7.9% (SD 1.4) [62.8 mmol/mol (SD 12.9)] to 6.9% (SD 1.2) [51.9 mmol/mol (SD 10.8)] in the traditional group, and 7.8% (SD 1.8) [61.7 mmol/mol (SD 17.2)] to 6.7% (SD 1.8) [49.7 mmol/mol (SD 17.3)] in the Internet group), with significant difference in the Internet group at the end of the study.

We did not find significant difference in TDD of insulin and weight change during the study. We noticed three DKA episodes in the Internet group (cannula occlusion and flu), in comparison with two DKA episodes in the traditional group (cannula occlusion), but there was no significant difference (Table 2). There were no severe hypoglycemia events in both groups.

**Table 1:** Clinical characteristics of patients enrolled in the study.

Patient characteristics		Traditional group	Internet group	*P* value
Number		29	27	
Age (years), mean (SD^a^)		16.9 (2.7)	17.4 (2.4)	.54
**Gender**				
	Male	13	12	
	Female	16	15	
Diabetes duration (years), mean (SD)		5.6 (2.1)	5.4 (2.8)	.92
BMI^b^, mean (SD)		22.4 (3.8)	21.7 (3.4)	.64

^a^SD: standard deviation.

^b^BMI: body mass index.

**Table 2:** Glucose control of patients before and at the end of study. Severe hypoglycemia and Diabetic Ketoacidosis (DKA) are calculated as total number of events during the study.

Glucose control	Baseline		After 3 years		
	Traditional group	Internet group	Traditional group	Internet group	*P* value
Hb_A1c_^a^ (%), mean (SD^b^)	7.9 (1.4)	7.8 (1.8)	6.9 (1.2)	6.7 (1.8)	<.5
Hb_A1c_ (mmol/mol), mean (SD)	62.8 (12.9)	61.7 (17.2)	51.9 (10.8)	49.7 (17.3)	<.5
Mean blood glucose (mmol/l), mean (SD)	9.7 (3.2)	9.8 (2.9)	8.8 (2.4)	8.6 (2.8)	<.5
TDD^c^ insulin (units), mean (SD)	48.6 (1.9)	45.4 (2.1)	51 (2.6)	49 (1.7)	NS^d^
Severe hypoglycemia			0	0	NS
DKA^e^			1	2	NS

^a^Hb_A1c_: glycosylated hemoglobin.

^b^SD: standard deviation.

^c^TDD: total daily insulin.

^d^NS: not significant.

^e^DKA: Diabetic Ketoacidosis.

## Discussion

This study evaluates Facebook as a tool for communication and treatment in type 1 diabetes patients on the insulin pump compared with traditional clinic visits.

Outside of the Internet, social media have been shown to improve disease management and health outcomes for patients [17,18]. The use of social media in health care has been widely advocated [19,20], but there is little evidence describing the current state of the science and whether or not these tools can be used to treat the patient and to evaluate the potential benefits [21]. A recent study shows that use of the CareLink system with regular upload and contact with a diabetes team is associated with significantly improved glycemic control in compliant patients on sensor augmented pump [22]. Our findings demonstrate that social media can be used as an Internet tool for treatment of type 1 diabetes patients on the insulin pump as a part of traditional clinic visits. The idea of a synergistic relationship between social media users is one of the main perceived advantages of using these platforms [23]. Some of the recent studies suggest that there is inappropriate substitution for in-person visits and can also potentially lead to harmful results [24]. CareLink analysis together with Facebook was used to advice the patients about their diabetes control and to make changes in basal rates, bolus wizard setting, adherence to therapy, approach for low and high blood sugar, and education. Personalization, presentation, and participation in social media and health care make them highly effective [25]. The content can be tailored to the priorities of the patients. Every Internet visit was personalized with the patient’s need (appropriate time and date) and used active patient participation in the decision-making process of diabetes management.

Despite the advantages of social media use in health, criticisms have emerged. The availability of misinformation is a risk, as health care providers are unable to control the content that is posted or discussed [26]. Our study tried to overcome these disadvantages, where the posted comments can be used if only the doctors granted the comment with “like.” Additionally, accepting new patients in the groups must pass several controls to be assured that they are real.

Several authors speculate and denounce the role of the Internet in diffusing the flow of disease-management information [27,28], which are reported in several studies with empirical data, where self-management and compliance to insulin pump therapy can be improved using telemedicine [29]. Our findings suggest that Facebook diabetes communities contain a plurality of participants, including patients, family members, and health care providers. Our patients share their personal experience about specific issues (eg, hypoglycemia treatment) from which others can gain information and knowledge. Facebook can be used as a motivational tool, where patients post their Hb_A1c_ levels, whereas others support them in the management of their diabetes. We can easily reach the patients with posting educational information on a Facebook group page. Acute complication (DKA and severe hypoglycemia events), TDD insulin, and weight change were not significant in both groups. Our study demonstrates a significant decrease (0.9%) in Hb_A1c_ in the Internet group after 3 years. One of the possible reasons for improved diabetes management can be addressed to Internet monthly visits.

The project was lead on a voluntary basis for both patients and doctors. We are trying to raise a new momentum in the possible treatment of type 1 diabetes using social media and understanding from health care decision makers to include this option in their services.

We found that by using social media, patients gain diabetes knowledge and information, can be closer to their health care providers, and interact in their daily adjustments and moreover, it could help patients cope better with their daily life. This trial suggests that patients with type 1 diabetes prefer to communicate with their health care providers using social media. Social media such as Facebook as a tool can assist in standard medical care to improve glucose control in a long-term period in adolescents with type 1 diabetes using an insulin pump therapy.
